# First-Principles Investigation of Zr-Based Equiatomic Quaternary Heusler Compounds Under Hydrostatic Pressure for Spintronics Applications

**DOI:** 10.3390/nano15231796

**Published:** 2025-11-28

**Authors:** Xiaoli Yuan, Sicong Liu, Peng Wan, Zhenjun Zhang, Chengjun Tao

**Affiliations:** College of Mechanics and Engineering Science, Hohai University, Nanjing 210024, China; 13204103150@163.com (S.L.); zhangzhenjun@hhu.edu.cn (Z.Z.); taochengjun@hhu.edu.cn (C.T.)

**Keywords:** quaternary Heusler alloys, magnetic properties, electronic properties, hydrostatic pressure, spin gapless semiconductors

## Abstract

The first-principles method using density functional theory (DFT) reveals the mechanics, electronic structure, and magnetic properties of six Zr-based equiatomic quaternary Heusler compounds and their transformation under hydrostatic pressure. The results show that these compounds maintain mechanical stability under hydrostatic pressures of 0–100 GPa, and the ductility of all the alloys is improved except ZrCrFeGe. In the ground state structure, ZrVFeAl and ZrCrFeGe are half metals, ZrVCoAl and ZrCrFeAl are spin gapless semiconductors, while ZrCrMnAl and ZrMnFeAl are regarded as nearly half metals. ZrVFeAl, ZrVCoAl, ZrCrFeAl, and ZrCrFeGe have high spin polarization and satisfy the Slater–Pauling rule, and their spin-flip band gaps are 0.43 eV, 0.35 eV, 0.14 eV, and 0.11 eV, respectively. These half-metallic compounds maintain half-metallicity within a certain pressure range, while spin gapless semiconductors (SGS) complete the SGS~half-metal~near-half-metal transition under hydrostatic pressure. These half-metallic compounds and spin gapless semiconductors are ideal candidates for spintronic applications.

## 1. Introduction

In recent years, hundreds of half-metallic materials have been discovered theoretically and experimentally by researchers, which has further promoted the development of spintronics. Among them, the progress of Heusler alloys is particularly remarkable [1]. Heusler alloys are a huge family in the alloys field. Classified from the types of elements contained, Heusler alloys can be divided into binary Heusler alloys, ternary Heusler alloys, including half-Heusler alloys, full Heusler alloys, and inverse Heusler alloys, and quaternary Heusler alloys (formulas: X_2_YZ, XYZ and XX′YZ, respectively, X/X′ and Y are normally transition metals or rare earth metals, while Z is from the main group category in the periodic table, typically an s-p metal.) [2,3]. Heusler alloys can be also classified into metallic Heusler alloys, half-metallic Heusler alloys, near-half-metallic Heusler alloys, and spingapless semiconductors (SGSs) if we classify them according to the strength of metal properties [2,3,4]. Many half-metallic materials and spin gapless semiconductors have been found in Heusler alloys, which are usually predicted as good spintronic materials [5,6]. The excellent performance of these materials comes from their attractive physical properties [7]. On the one hand, half-metallic Heusler alloys have a special electronic structure near the Fermi level, that is, their band structure behaves as a conductor in one spin channel and a semiconductor in the other spin channel; on the other hand, Heusler alloys usually have a high Curie temperature and are compatible with traditional semiconductors. The half-Heusler compound NiMnSb [8] is the first material predicted to be a half-metallic ferromagnet, reported by Groot et al. In addition to being used as spintronics materials, Heusler alloys also have many thought-provoking properties, such as shape memory effect [9,10], topology properties [11,12,13], optoelectronic, photovoltaic [14], superconductivity [15], thermoelectric properties [16,17] and so on. Near-half-metals (NHMs) are a special class of materials that exhibit electronic properties intermediate between metals and half-metals. To understand their classification criteria thoroughly, we need to examine their electronic structure, conductivity, and spin polarization. A near-half-metal is a material that is almost half-metallic but does not fully meet the strict criteria of a half-metal. A near-half-metal is classified based on its almost-but-not-fully spin-polarized electronic structure, with high yet incomplete spin polarization (70–99%) and a small but non-zero minority-spin DOS at E_F_. This distinguishes it from true half-metals (100% polarization) and conventional metals (no polarization). They are candidates for spintronics where ultra-high (but not perfect) spin polarization is acceptable. Small deviations from half-metallicity (due to defects, temperature, or strain) can turn a HM into an NHM. Their transport properties can be tuned by external fields, making them useful in spin valves and tunnel junctions [2]. Gao et al. [18] performed calculations for three types of these alloys after altering atoms positioning on the half-metallic property of quaternary Heusler alloys CoFeCrZ (Z = Al, Si, Ga, Ge). Accordingly, it was reported that CoFeCrGa and CoFeCrGe alloys (type 1) are nearly half-metals, but both CoFeCrAl and CoFeCrSi alloys (type 1) are energetically half-metallic ferromagnetism with half-metallic gaps of 0.16 and 0.28 eV, respectively [19].

In recent years, Wang et al. [20] proposed a zero-energy gap material that is expected to be used in spintronics devices, which is the so-called spin gapless semiconductor. The spin gapless semiconductors have only one spin channel that contributes to spin transport, and the other channel has an adjustable carrier concentration. It excites electrons from the valence band to the conduction band with only a very small amount of energy and has 100% spin polarization. Muhammad et al. [21] systematically studied the various properties of RhMnSb half-Heusler alloy under high pressure and believed that the alloy is suitable for spintronics or optics. Özdoğan et al. [22] used first principles to calculate 60 quaternary Heusler alloys and elaborated on the energy level structure of spin gapless semiconductors. Ma et al. [23] theoretically calculated 405 inverse Heusler alloys in the fourth period, and finally established 14 spin gapless semiconductors and 51 half-metals. Gao et al. [24] used high-throughput screening to screen dozens of stable spin gapless semiconductors from more than 10,000 quaternary Heusler compounds. In addition, quaternary Heusler alloys such as CoVRhGe [25], CoRuFeSi [26], NbVMnAl and NbFeCrAl [27] are theoretically predicted as spintronic materials.

Heusler compounds are promising candidates for spintronics due to their high spin polarization, tunable electronic structure, and compatibility with existing semiconductor technologies. However, Zr-based equiatomic quaternary Heusler compounds remain underexplored, particularly under hydrostatic pressure, which can significantly alter their electronic and magnetic properties. The quaternary Heusler alloy has the characteristics of low power consumption and high-performance flexibility [28]. Previous studies have focused on ternary Heusler alloys or non-equiatomic compositions, leaving the potential of equiatomic quaternary Zr-based systems (e.g., ZrAlFeZ where Z = Co, Cr, Ge, etc.) largely unknown. Moreover, pressure-tuning has been applied to other Heusler systems; its impact on spin polarization, bandgap engineering, and magnetic stability in Zr-based quaternary alloys remains unclear. In order to investigate and explore the properties of Zr-based quaternary Heusler alloys more systematically, this paper studies the stability, electronic structure, magnetic properties, and mechanical properties of ZrVFeAl, ZrCrMnAl, ZrVCoAl, ZrCrFeAl, ZrCrFeGe, ZrMnFeAl and their changes under high pressure.

## 2. Computational Details

The calculations in this paper are based on density functional theory (DFT), implemented in the Vienna ab initio simulation package (VASP) [29,30,31,32]. The interaction between atomic core and valence electrons is dealt with by using the projector augmented wave (PAW) method [33] which was more precise than the ultrasoft pseudopotentials. The generalized gradient approximation (GGA) of Perde–Burke–Ernzerhof (PBE) was used to express the exchange and correlation of electrons. The cut-off energy of the plane wave basis set was selected as 500 eV. The integral on the irreducible Brillouin zone (IBZ) uses the automatic grid generation scheme in VASP to generate a 15 × 15 × 15 Monkhorst–Pack grid [34]. All crystal structures are completely relaxed, and the conjugate gradient algorithm was used to optimize the position of atoms. The energy change in two consecutive ion steps is less than 1 × 10^−6^ eV, and the force on each ion converges within 0.02 eV/Å. In the calculation of spin-polarized GGA, the correlation part of the exchange correlation function adopts the Vosko–Wilk–Nusair interpolation method. In the calculation of electronic properties, we use the paths of Γ–X–W–K–Γ–L–U–W–L–K|U–X [2]. Table 1 shows the position of the symmetry K-points in the primitive reciprocal lattice.

## 3. Results and Discussions

### 3.1. Structural Stability

**Figure 1:**
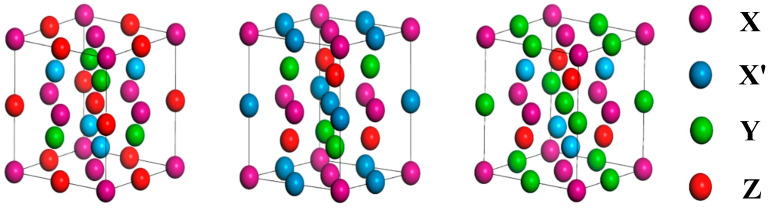
Crystal structure of equiatomic quaternary Heusler alloy for type-I, type-II, and type-III.

We investigated Heusler alloys, ternary Heusler alloys, and quaternary Heusler alloys. Among them, the space group of quaternary Heusler alloys is $F\bar{4}3m$ (No. 216) [2], which has three potential configurations. Table 2 shows three possible structures of quaternary Heusler alloys, and the three structure diagrams are shown in Figure 1. In order to find the ground state structure of these compounds, we performed spin-polarized structure relaxation calculations for all structures, looking for their natural lowest energy state, and found that the six compounds have the most stable structure when they are in the Y-I phase. Guo et al. used the CASTEP code to calculate the equilibrium lattice constant of ZrFeVAl as 6.22 Å [35], which is consistent with the calculation results in this paper. The equilibrium lattice constants of all materials in the ground state structure are listed in Table 4, and the formation energy is used to evaluate the structural stability of materials. Formation energy is an important condition for the thermodynamic stability of materials. Materials with negative formation energy are more likely to be synthesized. For the quaternary Heusler alloy, the calculation formula of the formation energy is [36]

(1) $$E_{f}=E_{XX^{\prime }YZ}-\left(E_{X}+E_{X^{\prime }}+E_{Y}+E_{Z}\right)$$

Here E_XX′YZ_ is the equilibrium total energy of each primitive cell of XX′YZ, while E_X_, E_X_′, E_Y_, and E_Z_ are the total energy of bulk X, X′, Y, and Z in the ground state structure, respectively. The formation energy of six Zr-based equiatomic quaternary Heusler compounds at the equilibrium lattice constants and experimental and theoretical calculation values of other similar complexes from other references as seen in Table 3. The results show that all compounds have low formation energy, which indicates that they can be synthesized. As indicated by the data comparison in Table 3, the formation energy of the complex we calculated is lower than that of other analogous complexes. A lower formation energy implies higher stability of the complex, which also demonstrates that the complex we calculated is more stable. On the other hand, this paper also examined the mechanical stability of these compounds through lattice dynamics calculations. The elastic constants are parameters that measure the strain produced by the crystal under stress. The cubic crystal system only has three independent elastic constants: C_11_, C_12_, and C_44_, which describe the mechanical properties and stability of the cubic crystal. The elastic stability criterion, also known as Born-Huang criteria [37], is given by the following formulas [38]:

(2) $$C_{11}-C_{12}>0,C_{11}+{2C}_{12}>0,C_{44}>0.$$

In fact, the calculated alloys all meet the Born–Huang criteria, which also confirms their stability from the mechanical point of view; future investigations incorporating phonon calculations would provide further insight into the dynamic stability of these compounds.

### 3.2. Elastic and Mechanical Properties

The bulk modulus B, shear modulus G, and Young’s modulus E of material are indicators to measure its hardness, resistance to plastic deformation, and stiffness, respectively, which can be obtained by performing some simple processing on the elastic constants [2,39,40,41,42,43]. All calculation results are shown in Table 4, and the Poisson’s ratio (ν), Pugh ratio (B/G), and Cauchy pressure (CP) of compounds are also given. Their formulas are as follows [42,43,44]:

(3) $$\nu =\frac{3B-2G}{2(3B+G)}$$

(4) $$CP=C_{44}-C_{12}$$

A Pugh ratio is proposed to judge whether the material is ductile or brittle. Ductility means the ability of a material to be drawn into a steel wire, while brittleness means that the material does not produce obvious plastic deformation under stress [44]. The threshold for judging ductility and brittleness is around 1.75 [44], and the Pugh ratio is greater than 1.75, which can be considered as the material has ductility, while the Pugh ratio of brittle materials is less than 1.75. Moreover, Cauchy pressure is usually used to judge this property, with a positive value representing ductility and a negative value implying brittleness [45]. Similarly, Poisson’s ratio can also reflect this property, and its threshold is about 0.25 [40]. This ratio also represents the bonding force and central force in the solid and judges the compressibility of the material. It can be observed from Table 4 that all six compounds have a certain degree of ductility, and ZrCrFeGe has the best ductility. The mechanical properties of these alloys will also be discussed later.

### 3.3. Electronic Structure and Magnetic Properties

**Figure 2:**
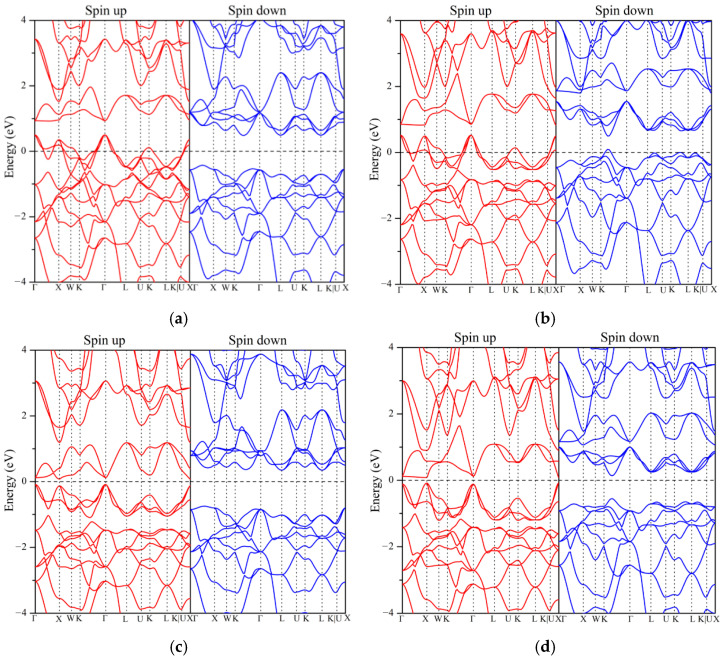

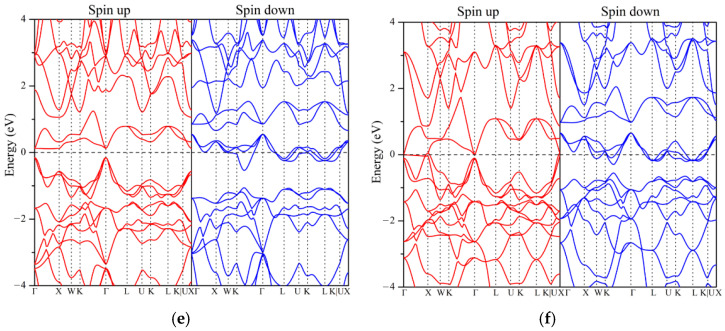
Spin-dependent band structure of these alloys at their equilibrium lattice constant. The corresponding relationship is (**a**) ZrVFeAl, (**b**) ZrCrMnAl, (**c**) ZrVCoAl, (**d**) ZrCrFeAl, (**e**) ZrCrFeGe, and (**f**) ZrMnFeAl.

**Figure 3:**
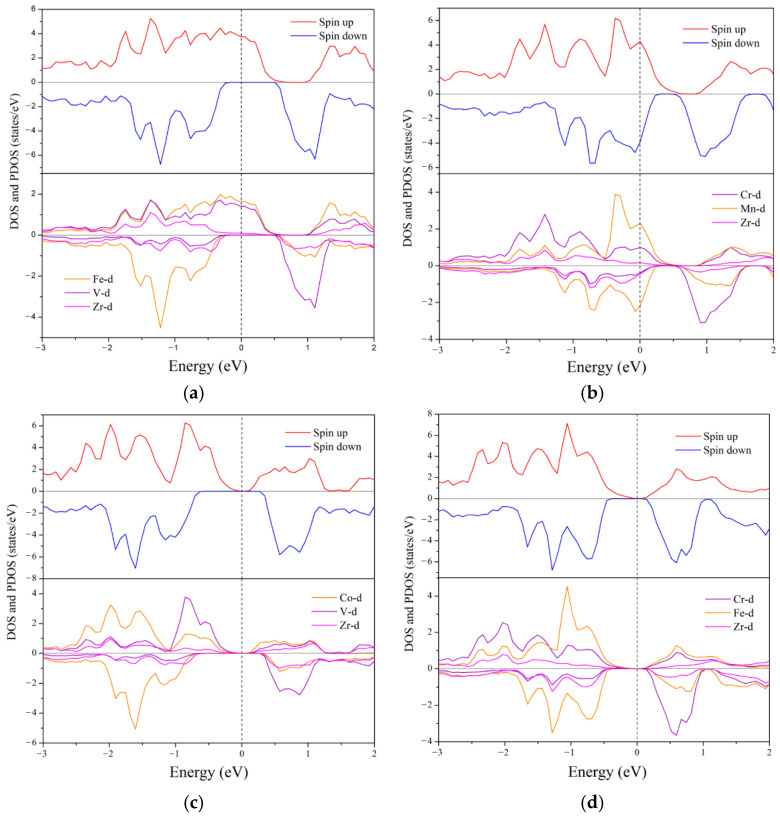

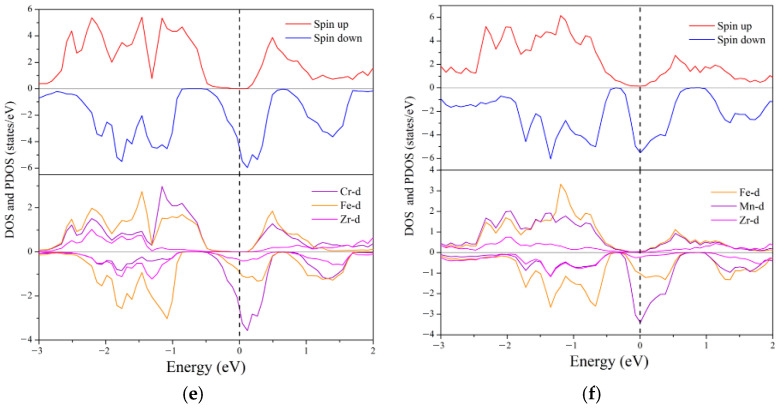
Total (TDOS) and partial density of states (PDOS) for Zr-based quaternary Heusler compounds at equilibrium constant. Fermi level is shown by vertical dashed line. The corresponding relationship is (**a**) ZrVFeAl, (**b**) ZrCrMnAl, (**c**) ZrVCoAl, (**d**) ZrCrFeAl, (**e**) ZrCrFeGe, and (**f**) ZrMnFeAl.

The macroscopic behavior of compounds often originates from their electronic structures. The calculation of electronic structures (including band structures and density of States) is helpful to define the properties of materials. Figure 2 and Figure 3 show the band structures and density of states of the six compounds in the ground state. According to the number of valence electrons of elements, the six alloys reported in this paper can be simply divided into three categories: 20 electrons system (ZrVFeAl, ZrCrMnAl), 21 electrons system (ZrVCoAl, ZrCrFeAl), and 22 electrons system (ZrCrFeGe, ZrMnFeAl). It can be clearly seen from the electronic structure of the compound that ZrVFeAl and ZrCrFeGe are half-metals, ZrVCoAl and ZrCrFeAl are spin gapless semiconductors, and ZrCrMnAl and ZrMnFeAl are near-half-metals. For these two kinds of near-half-metals, there is a gap at or near the Fermi level, which may be small enough to be called a pseudo gap. It is considered that when the pseudo gap is small enough or close enough to the Fermi level (within about 0.3 eV) [23], these compounds can be predicted as near-half-metals. From band structure diagrams, it can be seen that the two alloys of the 21 electrons system and ZrVFeAl with 20 electrons system have a band gap in the spin down channel, while the ZrCrFeGe with 22 electrons system has a band gap in the spin up channel. They look very similar on the whole, and it seems that the Fermi level rises with the total number of valence electrons, which is actually related to the change in the symmetry of compounds. It is worth mentioning that it is easier to find spin gapless semiconductors in 21, 26, and 28 electrons systems [24]. In addition, parameters such as the value of the band gap are listed in Table 3. The spin-flip band gap or half-metallic band gap is related to the stability of the compound’s half-metallicity. The small spin-flip band gap will cause the half-metallicity of materials to be destroyed by factors such as defects or temperature, which further affects their applications in spintronic devices [4]. Here, the spin-flip band gap is defined as the smaller of the energy at the top of the valence band and the energy at the bottom of the conduction band in the spin channel relative to the Fermi level [7]. ZrVFeAl, ZrVCoAl, ZrCrFeAl, and ZrCrFeGe all have large spin-flip band gaps, which are 0.43 eV, 0.35 eV, 0.14 eV, and 0.11 eV, respectively. It is generally considered that the value of the spin-flip band gap greater than 0.1 eV is fascinating, which can be seen from many reports [4].

The spin-flip band gap of ZrVFeAl reported by Guo et al. is 0.35 eV [30], which is consistent with the calculation results in this paper, and the difference is mainly due to the difference in the calculation code. The combination of perfect half-metallicity and a large spin-flip gap makes compounds like ZrVFeAl and ZrCrFeGe particularly promising candidates for spintronic applications. The 100% spin polarization at the Fermi level is a prerequisite for highly efficient spin injection. More importantly, the sizable spin-flip gaps (0.43 eV for ZrVFeAl and 0.11 eV for ZrCrFeGe) are critical for practical device performance. A large gap enhances the robustness of the half-metallic state against thermal fluctuations at room temperature and provides greater tolerance to defect-induced states that may arise during thin-film growth or from lattice mismatch with substrates. This intrinsic stability, coupled with their mechanical robustness as confirmed by the elastic criteria, positions these Zr-based Heusler alloys as compelling materials for next-generation spintronic devices such as magnetic tunnel junctions and spin filters. Table 5 also shows the magnetic parameters of the six compounds in the ground state. Magnetic research shows that, except for ZrCrMnAl and ZrMnFeAl, all other compounds satisfy the Slater–Pauling rule, which is also a key feature of half-metallic ferromagnets. The Slater–Pauling rule refers to the references [2,23]. Obviously, the ZrVFeAl of the 20 electrons system and the two SGS of the 21 electrons system as well as the ZrCrFeGe of the 22 electrons system satisfy the Slater–Pauling rule [2]. They all have the total magnetic moment in integer form and satisfy the Slater–Pauling rule perfectly. On the other hand, it can be found from Table 5 that the main-group element Z contributes very little to the total magnetic moment, which is consistent with many reports [7,25]. The total magnetic moment of compounds is mainly provided by Zr atom, X′ atom, Y atom. It is imprecise because the sum of the magnetic moments of each atom is not equal to the total magnetic moment. In fact, the cause of this situation is the difference in calculation methods.

The density of states of the compound can further reveal the key information such as the electronic hybridization of each element to help us better understand its electronic structure. For the two semi-metals and the two SGS, in view of the band structure, they all have a large band gap in the spin channel that behaves as a semiconductor, and their energy gap values are also listed in Table 5. The main-group element Z and the contribution of s and p orbitals to the total density of states are negligible, so they are not shown. Spin polarization P of any compounds is a way to describe their spin injection efficiency. A high degree of spin polarization can increase the concentration of spin carriers and achieve effective spin injection. The formula for calculating the spin polarization P is as follows [44]:

(5) $$P=\frac{DOS\left(E_{F}\left(↑\right)\right)-DOS(E_{F}(↓))}{DOS\left(E_{F}\left(↑\right)\right)+DOS(E_{F}(↓))}\times 100\%$$

**Figure 4:**
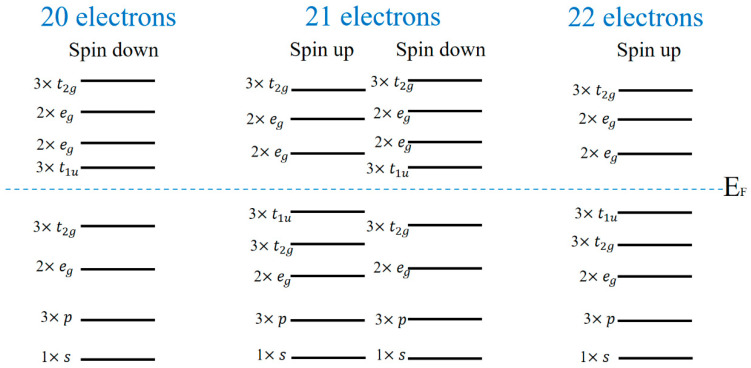
Schematic representation of the spinup and spindown band structure energy levels of the 20, 21 and 22 electrons systems in the Zr-based quaternary Heusler compounds.

In this formula, the values of DOS(E_F_(↑)) and DOS(E_F_(↓)) are both taken at the Fermi level. Both two half-metallic alloys and two SGS have 100% spin polarization. For nearly half-metallic materials, the spin up channel of ZrMnFeAl has a pseudo gap at the Fermi level, so it also has a spin polarization of 95%. In addition, it can be seen from the density of states that there is strong electron hybridization near the Fermi level, which is mainly provided by the d orbitals of the three transition metal elements. The similar electronic structure implies that they have similar energy levels and hybridization processes, and the half-metallic band gap of compounds originated here. The d orbital of X′ element hybridizes and produces 5 bond hybrids (2 × e_g_ and 3 × t_2g_). These hybrids are further hybridized with the d orbital of Zr element, resulting in 5 bond hybrids and 5 anti-bond hybrids (2 × e_u_ and 3 × t_1u_). Then the synthesized state of X′-Zr is hybridized with the d state of Y, forming 5 bonding states and 5 anti-bonding states. From the calculation results of the electronic structure, due to the influence of symmetry, the Fermi level also moves with the increase in the total valence electrons, which is specifically manifested as the Fermi level moves toward the original higher energy level. Figure 4 is a schematic diagram of the energy level structure of these compounds, and the report by Özdoğan et al. also shows this phenomenon [22].

### 3.4. Pressure Effect

In this paper, the physical properties of these compounds under hydrostatic pressure are calculated. The main motivation of this research comes from the fact that the synthesis of corresponding materials in experiments can easily lead to changes in the lattice constants (mainly including the imperfect match of the substrate lattice selected for the growth of the film and the defects that are difficult to control) and fascinating changes in the physical properties of the bulk under high pressure. We mainly evaluated the mechanical stability, half-metallicity, and magnetic properties of these compounds under high pressure. Figure 4 shows the change trend of elastic constants of six alloys under hydrostatic pressure (0–100 GPa). Obviously, these compounds meet the Born–Huang criteria in the range of 0–100 GPa and maintain mechanical stability. Furthermore, with the continuous increase in pressure, the bulk modulus, shear modulus, and Young’s modulus all change with the change in elastic constant. The specific performance is the following: except ZrCrMnAl in the range of 90–100 GPa, the bulk modulus, shear modulus, and Young’s modulus of all alloys increase with the increase in pressure. These results indicate that these alloys have a strong covalent bond as the bulk modulus increases, and because the bulk modulus is greater than the shear modulus, they are more difficult to deform under low pressure, while the shear modulus value is small under low pressure, which implies that these alloys are more suitable for processing at lower pressures. In addition, Young’s modulus enlargements with increasing pressure, which means that these alloys are harder under high pressure. As we mentioned earlier, these compounds have a certain degree of ductility at zero pressure, and under high pressure, the ductility of these compounds also changes. The ductility of ZrCrFeGe decreases with increasing pressure. The ductility of ZrCrMnAl is getting larger and larger in the range of 0–90 GPa and is reduced due to the sudden change in elastic constants under 90–100 GPa. The ductility of other alloys increases with increasing pressure. The parameters of mechanical properties under high pressure can be found in Appendix A.

**Figure 5:**
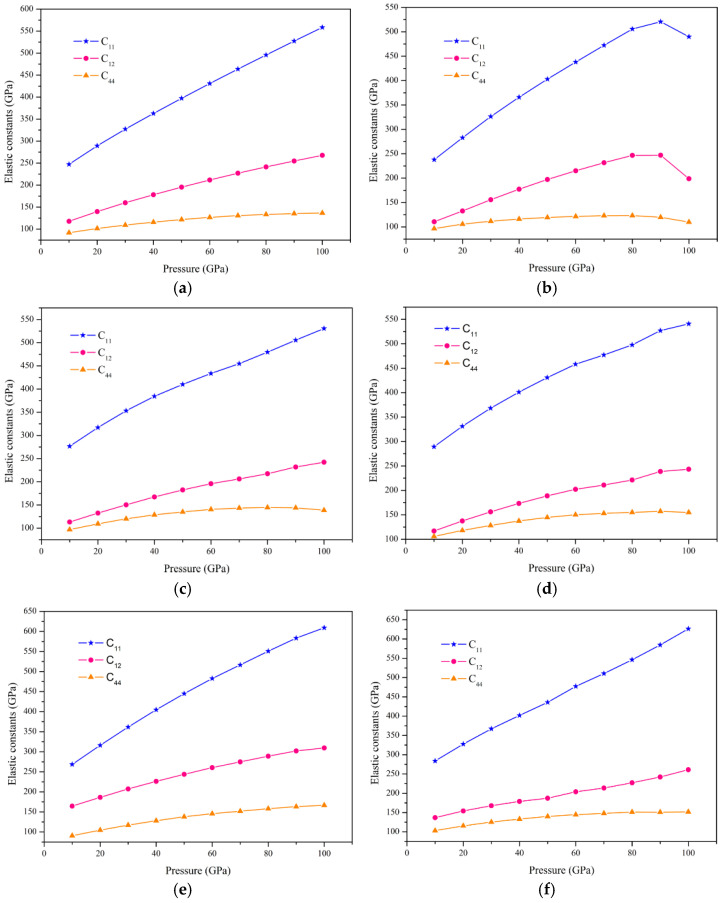
The effect of high pressure on elastic constants of six Zr-based quaternary Heusler compounds. The corresponding relationship is (**a**) ZrVFeAl, (**b**) ZrCrMnAl, (**c**) ZrVCoAl, (**d**) ZrCrFeAl, (**e**) ZrCrFeGe, and (**f**) ZrMnFeAl.

**Figure 6:**
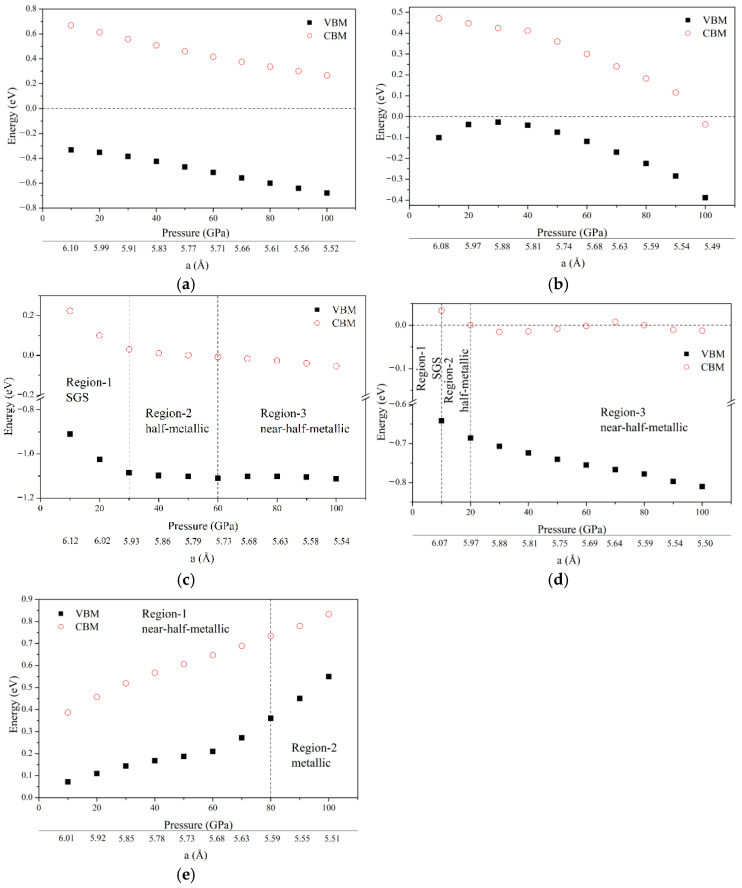
The valence band maximum (VBM) and conduction band minimum (CBM) of Zr-based quaternary Heusler compounds under different pressures are obtained. The corresponding relationship is (**a**) ZrVFeAl, (**b**) ZrCrMnAl, (**c**) ZrVCoAl, (**d**) ZrCrFeAl, and (**e**) ZrCrFeGe.

This paper also theoretically explored the influence of pressure on the electronic structure and magnetic properties of the compound, and the results are shown in Figure 5 and Figure 6. For the 20 electrons system, both ZrVFeAl and ZrCrMnAl maintain half-metallicity under high hydrostatic pressure (0–100 GPa, 0–90 GPa, respectively) or lattice strain (6.24–5.52 Å, 6.20–5.54 Å, respectively), and abide by the Slater–Pauling rule. The Fermi level moves to the originally higher energy state. In addition, the total magnetic moment of the alloys is mainly affected by the atomic magnetic moments of the X′ and Y elements, and the atomic magnetic moments of Zr and the main-group element Z change slightly under high pressure. The good stability of half-metallicity under large lattice strain indicates that they will perform well in practical applications (such as electrodes of magnetoresistive junctions [46], etc.) and are not easily disturbed.

**Figure 7:**
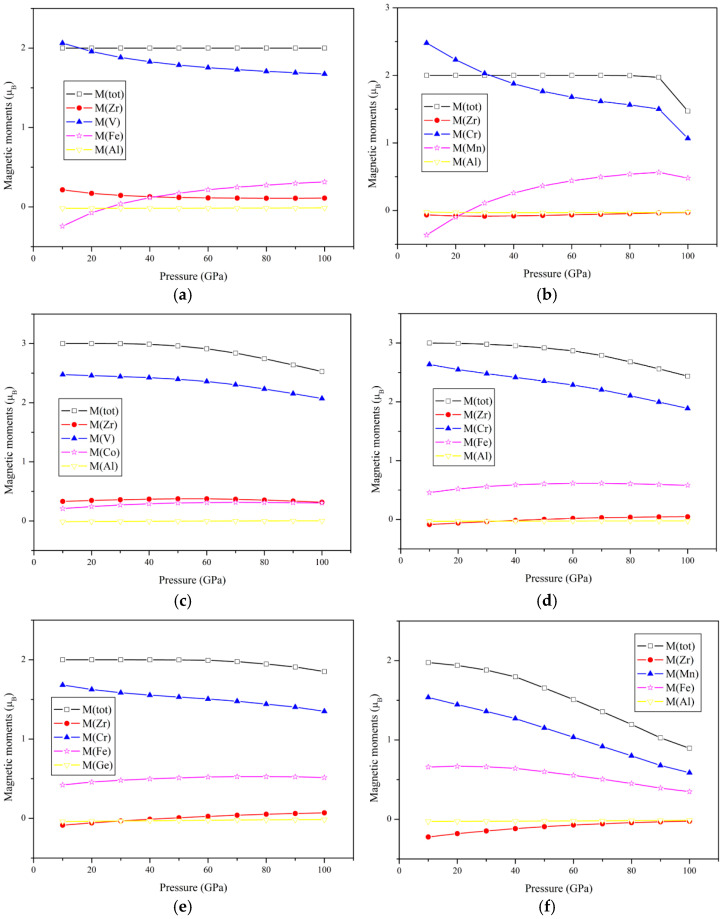
Total and atomic spin magnetic moment of Zr-based quaternary Heusler alloys at different pressures. The corresponding relationship is (**a**) ZrVFeAl, (**b**) ZrCrMnAl, (**c**) ZrVCoAl, (**d**) ZrCrFeAl, (**e**) ZrCrFeGe, and (**f**) ZrMnFeAl.

For the 22 electrons system, at zero pressure, ZrCrFeGe has an energy gap of 0.26 eV in the spin up channel, but as the pressure increases, the Fermi level moves to the original lower energy state, and the energy gap quickly penetrates the Fermi level, and this causes ZrCrFeGe to transform into a nearly half-metallic material under the hydrostatic pressure of 10 GPa (6.01 Å) (Region-1) (the standard is whether the energy gap mentioned above is close enough or small enough to the Fermi level; this value is about 0.3 eV), and as the pressure further increases to 80 GPa (5.59 Å) (Region-2), ZrCrFeGe can be considered as a transition from nearly half-metal to metal. Since ZrMnFeAl has only a small pseudo-gap at the Fermi level under zero pressure, it is already a nearly half-metallic material in the ground state structure, which also causes the spin polarization to be less than 100%. For the above reasons (there is no real gap), we did not give a diagram of the influence of hydrostatic pressure on VBM and CBM. The half-metal of ZrMnFeAl is also weak, and it changes from half-metal to metal at 10 GPa (6.00 Å). The absolute value of the total magnetic moment of ZrCrFeGe and ZrMnFeAl both decrease with the increase in hydrostatic pressure (Figure 7). Here, for the convenience of comparison, we have performed the inverse treatment of the magnetic moment. It can be seen that ZrCrFeGe obeys the Slater–Pauling rule in a large range (0–80 GPa, or 6.12–5.59 Å) because it also maintains half-metallicity in this range. ZrCrFeGe is a half-metallic alloy that is more suitable for low-pressure applications.

For the 21 electrons system, they have a more complex electronic structure transition. Both ZrVCoAl and ZrCrFeAl have undergone a transformation process from SGS (Region-1)-half-metal (Region-2)-near-half-metal (Region-3). ZrVCoAl is still a spin gapless semiconductor under pressure of 0–30 GPa (6.25–5.93 Å) and then turns into a half-metallic material because the spin up channel passes through the Fermi level. A 60 GPa (5.73 Å) is the critical value for the transition from half-metal to nearly half-metal. ZrCrFeAl is rapidly transformed from SGS to half-metallic material. It is SGS in the region of 0–10 GPa (6.19–6.07 Å) and maintains half-metallic property in the region of 10–20 GPa (6.07-5.97 Å). After that, it shows nearly half-metallic behavior in a large range (20–100 GPa or 5.97-5.50 Å). The reason for such a large electronic structure change within such a small stress–strain range is due to the narrower energy gap in the ZrCrFeAl spin up channel, which makes ZrCrFeAl sensitive to lattice strain changes. The variation trend of their total magnetic moment under high pressure is similar to the two compounds of the 20 electrons system, and the change in the total magnetic moment is more affected by the change in the atomic magnetic moments of X′ and Y.

As mentioned above, the large spin-flip band gap can improve the half-metallic stability of the alloy. Under hydrostatic pressure, the half-metallicity of ZrVFeAl is least affected by the stress–strain effect. In addition, for the six compounds discussed in this article, the band gap in the spin down channel is larger than the band gap in the spin up channel, and the alloys are more likely to have a large spin-flip band gap and stable semi-metallicity. While our computational approach provides valuable insights, several limitations must be acknowledged: the use of GGA-PBE may underestimate electron correlation effects, potentially affecting bandgap predictions. Hybrid functionals or GW corrections could improve accuracy but at higher computational cost. Neglecting Spin–Orbit Coupling in 4d/3d systems (e.g., Zr, Cr) might underestimate magnetic anisotropy or spin-flip gaps, critical for device stability. Real-world samples often exhibit atomic disorder which could degrade spin polarization-a factor not modeled here; for example, experimental studies of similar quaternary Heusler report ~70–80% spin polarization due to disorder, vs. our ideal 100% prediction. Our calculations assume T = 0 K, ignoring thermal fluctuations that may reduce T_C_ or destabilize HM/SGS states at ambient conditions. Although extreme pressures (e.g., 100 GPa) are beyond device operating conditions, they establish theoretical limits of electronic phase stability. Critically, transitions below 30 GPa (e.g., 30 GPa in ZrVCoAl ≈ 5% strain) guide strain engineering strategies for thin-film spintronics [47]. The persistence of half-metallicity in ZrVFeAl/ZrCrMnAl under large compression further highlights their potential for robust device design.

It is important to note that the present study, conducted at zero temperature and without considering spin–orbit coupling, provides a foundational understanding. Future work incorporating these effects will be crucial to assess the operational stability of the half-metallic state under realistic conditions.

## 4. Conclusions

This study investigates the structural, electronic, and magnetic properties of six Zr-based equiatomic quaternary Heusler alloys (ZrVFeAl, ZrCrMnAl, ZrVCoAl, ZrCrFeAl, ZrCrFeGe, ZrMnFeAl) under hydrostatic pressure using first-principles calculations. Key findings are the following:

1. Ground State Properties: At equilibrium, ZrVFeAl and ZrCrFeGe are half-metals (HMs), ZrVCoAl and ZrCrFeAl are spin gapless semiconductors (SGSs), while ZrCrMnAl and ZrMnFeAl are near-HMs. All exhibit integer total magnetic moments, satisfying the Slater–Pauling rule, and possess 100% spin polarization with significant spin-flip gaps.
2. Pressure-Induced Transformations: Under pressure (0–100 GPa), all alloys remain mechanically stable (Born–Huang criteria). Crucially, ZrVFeAl and ZrCrMnAl retain HM character over their entire studied pressure ranges (0–100 GPa and 0–90 GPa, respectively). ZrVCoAl transitions from SGS to HM at ~30 GPa. ZrCrFeAl evolves from SGS (0–10 GPa) to HM (10–20 GPa), becoming near-HM at higher pressures.
3. Mechanical Behavior: All studied alloys demonstrate ductility, suggesting favorable processability particularly at lower pressures.

These results highlight the tunable electronic structure of Zr-based quaternary Heuslers under pressure, identifying specific compounds (notably ZrVFeAl, ZrCrMnAl, and pressure-tuned ZrVCoAl/ZrCrFeAl) as robust or pressure-induced HMs/SGSs with potential for spintronic applications. Moreover, our current study focused on the ground state, lays a solid foundation for future investigations. To bridge the gap between ideal predictions and practical applications, subsequent work should prioritize the inclusion of spin-orbit coupling and finite-temperature effects to rigorously evaluate the operational stability of these materials under realistic conditions.

## Figures and Tables

**Table 1:** Symmetry k-points of FCC lattice.

	s_1_	s_2_	s_3_		s_1_	s_2_	s_3_
Γ	0	0	0	K	0.375	0.375	0.75
X	0.5	0	0.5	L	0.5	0.5	0.5
W	0.5	0.25	0.75	U	0.625	0.25	0.625

**Table 2:** The three atomic occupations of the quaternary Heusler compound XX′YZ (space group $F\bar{4}3m$).

Type	X	X′	Y	Z
Y-I	4a(0,0,0)	4c(0.25,0.25,0.25)	4d(0.75,0.75,0.75)	4b(0.5,0.5,0.5)
Y-II	4a(0,0,0)	4b(0.5,0.5,0.5)	4c(0.25,0.25,0.25)	4d(0.75,0.75,0.75)
Y-III	4a(0,0,0)	4c(0.25,0.25,0.25)	4b(0.5,0.5,0.5)	4d(0.75,0.75,0.75)

**Table 3:** Calculated formation energies (in eV) for six Zr-based equiatomic quaternary Heusler compounds at the equilibrium lattice constants vs. experimental and theoretical calculation values of other similar complexes from other references.

Complexes (Our Work)	Types	Formation Energy E_f_ (eV)	Other Similar Complexes	Formation Energy E_f_ (eV)
ZrVFeAl	Y-I	−1.37	CoFeCrAl [18]	−0.86
ZrCrMnAl	Y-I	−0.33	CoFeCrGa [18]	−0.52
ZrVCoAl	Y-I	−1.54	CoFeCrSi [18]	−1.48
ZrCrFeAl	Y-I	−1.33	CoFeCrGe [18]	−0.66
ZrCrFeGe	Y-I	−0.97	CoFeMnAl [18]	−1.31
ZrMnFeAl	Y-I	−1.55	CoFeMnGa [18]	−0.82
	Y-I		CoFeMnSi [18]	−1.96
			CoFeMne [18]	−1.15

**Table 4:** The calculated equilibrium lattice constants a (Å), elastic constants C_11_, C_12_, C_44_ (GPa), bulk modulus B (GPa), shear modulus G (GPa), Young’s modulus E (GPa), Poisson’s ratio ν, Pugh ratio (B/G), and Cauchy pressure CP (GPa) of six Zr-based equiatomic quaternary Heusler compounds.

Alloys	ZrVFeAl	ZrCrMnAl	ZrVCoAl	ZrCrFeAl	ZrCrFeGe	ZrMnFeAl
state	Y-I	Y-I	Y-I	Y-I	Y-I	Y-I
a	6.24	6.20	6.25	6.19	6.12	6.11
C_11_	198.74	207.23	229.19	241.28	218.25	227.17
C_12_	92.77	96.84	91.35	93.60	139.80	115.40
C_44_	79.49	80.15	81.16	90.35	74.28	89.01
B	128.10	133.64	137.29	142.83	165.95	152.65
G	67.56	69.02	76.02	83.34	57.49	73.86
E	172.38	176.65	192.53	209.31	154.61	190.80
ν	0.28	0.28	0.27	0.27	0.35	0.29
B/G	1.90	1.94	1.81	1.71	2.89	2.07
CP	13.27	16.7	10.19	3.25	65.52	26.39

**Table 5:** The calculated total and atom resolved magnetic moments (μ_B_), energy gap E_g_ (eV), spin-flip band gap E_HM_ (eV), spin polarization P and physical nature of six Zr-based equiatomic quaternary Heusler alloys.

Alloys	M_tot_	M_Zr_	M_X′_	M_Y_	M_Z_	E_g_	E_HM_	P	Physical Nature
ZrVFeAl	2.00	0.29	2.22	−0.52	−0.02	0.91	0.43	100%	half-metal
ZrCrMnAl	2.58	−0.18	2.43	0.37	−0.04	-	-	5%	near-half-metal
ZrVCoAl	3.00	0.31	2.50	0.16	−0.02	1.10	0.35	100%	SGS
ZrCrFeAl	3.00	−0.11	2.75	0.36	−0.03	0.69	0.14	100%	SGS
ZrCrFeGe	−2.00	0.12	−1.77	−0.36	0.05	0.26	0.11	100%	half-metal
ZrMnFeAl	−2.02	0.28	−1.67	−0.63	0.03	-	-	95%	near-half-metal

## Data Availability

Data will be made available on request.

## Appendix A

The mechanical properties of compounds under hydrostatic pressure are presented in Table A1, Table A2, Table A3, Table A4, Table A5 and Table A6.

**Table A1:** ZrVFeAl.

Pressure (GPa)	B	G	E	ν	CP	B/G
10	160.95	79.84	205.53	0.29	25.94	2.02
20	189.71	89.68	232.41	0.30	38.59	2.12
30	215.66	98.15	255.66	0.30	50.71	2.20
40	239.73	105.81	276.71	0.31	62.30	2.27
50	262.70	112.97	296.42	0.31	73.62	2.33
60	284.71	119.58	314.69	0.32	84.90	2.38
70	305.92	125.62	331.49	0.32	96.31	2.44
80	326.19	130.88	346.30	0.32	107.93	2.49
90	345.65	135.65	359.87	0.33	119.56	2.55
100	364.64	140.05	372.46	0.33	131.11	2.60

**Table A2:** ZrCrMnAl.

Pressure (GPa)	B	G	E	ν	CP	B/G
10	153.08	81.74	208.17	0.27	14.01	1.87
20	182.73	92.19	236.76	0.28	26.92	1.98
30	212.64	100.20	259.79	0.30	44.19	2.12
40	240.21	106.89	279.26	0.31	61.12	2.25
50	265.79	112.50	295.78	0.32	77.79	2.36
60	289.44	117.40	310.24	0.32	93.61	2.47
70	311.83	121.97	323.71	0.33	108.53	2.56
80	333.00	125.73	335.03	0.33	123.39	2.65
90	338.25	126.34	337.05	0.33	127.24	2.68
100	295.82	123.03	324.15	0.32	88.85	2.41

**Table A3:** ZrVCoAl.

Pressure (GPa)	B	G	E	ν	CP	B/G
10	167.79	90.45	230.02	0.27	16.52	1.86
20	194.03	102.18	260.77	0.28	23.14	1.90
30	218.00	112.33	287.59	0.28	30.14	1.94
40	239.72	120.20	308.95	0.29	38.70	1.99
50	258.40	126.11	325.40	0.29	47.47	2.05
60	275.19	131.43	340.14	0.29	55.41	2.09
70	289.08	135.52	351.61	0.30	62.67	2.13
80	304.85	139.05	362.09	0.30	72.83	2.19
90	323.09	141.08	369.47	0.31	87.88	2.29
100	338.42	140.87	371.12	0.32	103.59	2.40

**Table A4:** ZrCrFeAl.

Pressure (GPa)	B	G	E	ν	CP	B/G
10	174.36	97.48	246.50	0.26	11.08	1.79
20	202.02	109.10	277.37	0.27	19.33	1.85
30	226.78	118.82	303.47	0.28	27.89	1.91
40	249.17	127.50	326.76	0.28	35.76	1.95
50	269.44	134.67	346.32	0.29	44.13	2.00
60	287.54	140.83	363.19	0.29	52.10	2.04
70	299.60	144.74	373.99	0.29	57.80	2.07
80	313.42	148.05	383.73	0.30	66.29	2.12
90	334.73	151.85	395.70	0.30	81.43	2.20
100	342.43	152.43	398.21	0.31	88.33	2.25

**Table A5:** ZrCrFeGe.

Pressure (GPa)	B	G	E	ν	CP	B/G
10	199.14	72.53	194.04	0.34	73.80	2.75
20	229.55	86.43	230.39	0.33	81.56	2.66
30	258.79	99.08	263.60	0.33	90.22	2.61
40	285.70	110.86	294.49	0.33	98.05	2.58
50	310.83	121.48	322.44	0.33	105.94	2.56
60	334.47	130.73	346.98	0.33	114.67	2.56
70	355.37	138.58	367.92	0.33	122.95	2.56
80	376.31	146.54	389.10	0.33	131.04	2.57
90	395.83	153.83	408.56	0.33	138.76	2.57
100	409.42	159.74	424.08	0.33	142.82	2.56

**Table A6:** ZrMnFeAl.

Pressure (GPa)	B	G	E	ν	CP	B/G
10	185.83	90.02	232.51	0.29	33.73	2.06
20	211.91	102.90	265.70	0.29	38.74	2.06
30	234.22	114.37	295.08	0.29	42.40	2.05
40	253.18	123.99	319.76	0.29	45.77	2.04
50	270.07	133.56	343.97	0.29	47.09	2.02
60	294.98	141.41	365.77	0.29	59.21	2.09
70	312.53	148.14	383.79	0.29	65.63	2.11
80	333.54	154.52	401.55	0.30	75.92	2.16
90	356.34	158.77	414.72	0.31	91.22	2.24
100	382.87	163.52	429.43	0.31	109.21	2.34
